# Is “cooling then freezing” a humane way to kill amphibians and reptiles?

**DOI:** 10.1242/bio.012179

**Published:** 2015-05-26

**Authors:** Richard Shine, Joshua Amiel, Adam J. Munn, Mathew Stewart, Alexei L. Vyssotski, John A. Lesku

**Affiliations:** 1 School of Biological Sciences A08, University of Sydney, Sydney, New South Wales 2006, Australia; 2 Institute for Conservation Biology and Environmental Management, School of Biological Sciences, University of Wollongong, Wollongong, New South Wales 2522, Australia; 3 Institute of Neuroinformatics, University of Zurich/ETH Zurich, Winterthurerstrasse 190, CH-8057 Zurich, Switzerland; 4 School of Life Sciences, La Trobe University, Melbourne, Victoria 3086, Australia

**Keywords:** Animal welfare, *Bufo marinus*, Ectothermy, Evidence-based practice

## Abstract

What is the most humane way to kill amphibians and small reptiles that are used in research? Historically, such animals were often killed by cooling followed by freezing, but this method was outlawed by ethics committees because of concerns that ice-crystals may form in peripheral tissues while the animal is still conscious, putatively causing intense pain. This argument relies on assumptions about the capacity of such animals to feel pain, the thermal thresholds for tissue freezing, the temperature-dependence of nerve-impulse transmission and brain activity, and the magnitude of thermal differentials within the bodies of rapidly-cooling animals. A review of published studies casts doubt on those assumptions, and our laboratory experiments on cane toads (*Rhinella marina*) show that brain activity declines smoothly during freezing, with no indication of pain perception. Thus, cooling followed by freezing can offer a humane method of killing cane toads, and may be widely applicable to other ectotherms (especially, small species that are rarely active at low body temperatures). More generally, many animal-ethics regulations have little empirical basis, and research on this topic is urgently required in order to reduce animal suffering.

## INTRODUCTION

Concern about the ethical treatment of animals has prompted extensive discussion of how to minimise suffering. Unfortunately, human intuition may fail to predict the stress and suffering of species that are only distantly related to us (Rose, 2002, 2007; Stevens, 2004; Langkilde and Shine, 2006). For example, mammals and birds typically react to falling ambient temperatures by attempting to maintain body temperature (by increasing metabolic heat production). Thus, exposure to low temperatures may cause intense discomfort. In contrast, many amphibians and reptiles exhibit highly variable body temperatures in the course of their day-to-day lives, and react to falling temperatures by becoming torpid (Pough, 1980; Frankenhaeuser and Moore, 1963; Rosenberg, 1978; Roberts and Blackburn, 1975; LaManna et al., 1980; Hunsaker and Lansing, 1962). Periods of low body temperature associated with inactivity are common on a seasonal or even daily basis for many ectotherms, even for species (such as lowland tropical taxa, and diurnal heliotherms) that spend most of their activity time at relatively high body temperatures (Pough, 1980).

The immobility and unresponsiveness of such “high-temperature” ectotherms at low ambient temperatures suggest that their brain activity is reduced when they are cold. If that is true, then such animals could be humanely killed by cooling them to induce torpor (to reduce brain activity and thus, pain perception); and then reducing their temperature even further, to lethal levels. This was a popular method for humane killing of experimental animals for many years (McDonald, 1976), widely endorsed by animal welfare organisations (NSW Animal Welfare Advisory Council, 2004). However, opinion has shifted. Globally, modern veterinary guidelines now rule that “cooling then freezing” is ethically unacceptable (e.g. https://www.avma.org/kb/policies/documents/euthanasia.pdf). Ethics guidelines rarely cite primary research literature (Martin, 1995), and the argument against “cooling then freezing” rests upon a hypothesis rather than specific empirical data. The hypothesis is that temperatures low enough to induce ice-crystal formation in peripheral tissues are nonetheless high enough to allow painful sensations to travel through peripheral nerves and reach the brain, which in turn is warm enough to register those sensations as painful (Sharp et al., 2011). The plausibility of this scenario depends upon the temperatures at which ice crystals form relative to the temperatures at which nerves and brains cease to function; and on the magnitude of thermal differentials within an animal's body during rapid cooling.

To evaluate this scenario, we (a) reviewed published literature on thermal dependency of nervous-system function to examine the assumptions that have outlawed “cooling then freezing”, and (b) measured brain activity and limb-core thermal differentials directly in ectotherms while they were being frozen. As a case study, we used the cane toad (*Rhinella marina*). These invasive anurans are spreading across Australia (Urban et al., 2007), fatally poisoning native predators (Shine, 2010). Community “toad-busting” groups kill many thousands of toads annually, by a variety of methods – some of which appear to be cruel (Clarke et al., 2009) or unreliable (Sharp et al., 2011); toads are also killed for university teaching and research. The prohibition on “cooling then freezing” has outlawed the most readily accessible method for killing ectotherms: for example, current guidelines for euthanasia of cane toads recommend blunt trauma or decapitation (Sharp et al., 2011), methods poorly suited to use by untrained people. Thus, the cane toad offers an excellent example of why it is important to know whether or not the prohibition of euthanasia via hypothermia is based on rational grounds.

## RESULTS

### Review of published literature

There is significant scientific debate about whether or not ectothermic vertebrates experience “pain” in the way that humans understand the term; several authorities suggest that we should talk of “nociception” instead (Rose, 2002, 2007; Stevens, 2004). Even if a noxious stimulus induces activity in the brain, the process may not involve anything comparable to “pain” as perceived by humans (Key, 2015). Nonetheless, although the subjective experience of a cane toad may be very different to that of a human, most animal ethics committees (and the wider community) continue to believe that amphibians can feel pain.

Even if we accept the contested point that an amphibian is capable of feeling pain, our review of published literature does not support the idea that placing a pre-cooled ectotherm into a freezer is inhumane, for at least four reasons:

1. When a small ectotherm is pre-cooled and then placed into a freezer, thermal differentials within its body are minor (Hillman et al., 2009; Wilson et al., 2009; Bicego et al., 2007) and thus, the animal's brain cools almost as rapidly as do its limbs. Its neural network is likely to be close to freezing by the time that ice crystals form in peripheral tissues.
2. Small animals cool to well below 0°C before freezing begins, allowing time for deep-body temperatures to fall to low levels. In anurans, ice crystals form at −1 to −4.3°C (Hillman et al., 2009). Thus, the critical issue is whether peripheral nerves can transmit nociceptive signals when the superficial tissue of the animal falls below −1°C. In ectotherms, transmission velocities for nerve impulses fall rapidly at low temperatures (Frankenhaeuser and Moore, 1963), and cease at temperatures close to 0°C: for example, 1.3–4°C in tortoises (Rosenberg, 1978), 0–2°C in frogs (Roberts and Blackburn, 1975). In frogs, the nociceptive peripheral neurons cease functioning at higher temperatures than do those transmitting signals such as touch or proprioception (Roberts and Blackburn, 1975).
3. Cold is an anaesthetic (Wilson et al., 2009). Even a modest reduction in skin temperature reduces painful sensations in mammals (Bicego et al., 2007). In amphibians, minor cooling of one limb (to 6°C) reduces the animal's reaction to noxious stimuli (Suckow et al., 1999). In cane toads, low temperatures have the same structural and electrophysiological effects on myelinated nerves as do local anaesthetics such as lidocaine (Luzzati et al., 1999). Thus, nerve endings close to the peripheral tissues being frozen are unlikely to transmit nociceptive (“pain”) signals.
4. Normal brain functioning is dependent upon temperature in ectotherms. The brains of cane toads fail to respond to electrical stimulation below 3.2°C (LaManna et al., 1980), broadly similar to many other ectotherms (Hunsaker and Lansing, 1962).

### Experimental study

**Fig. 1.**
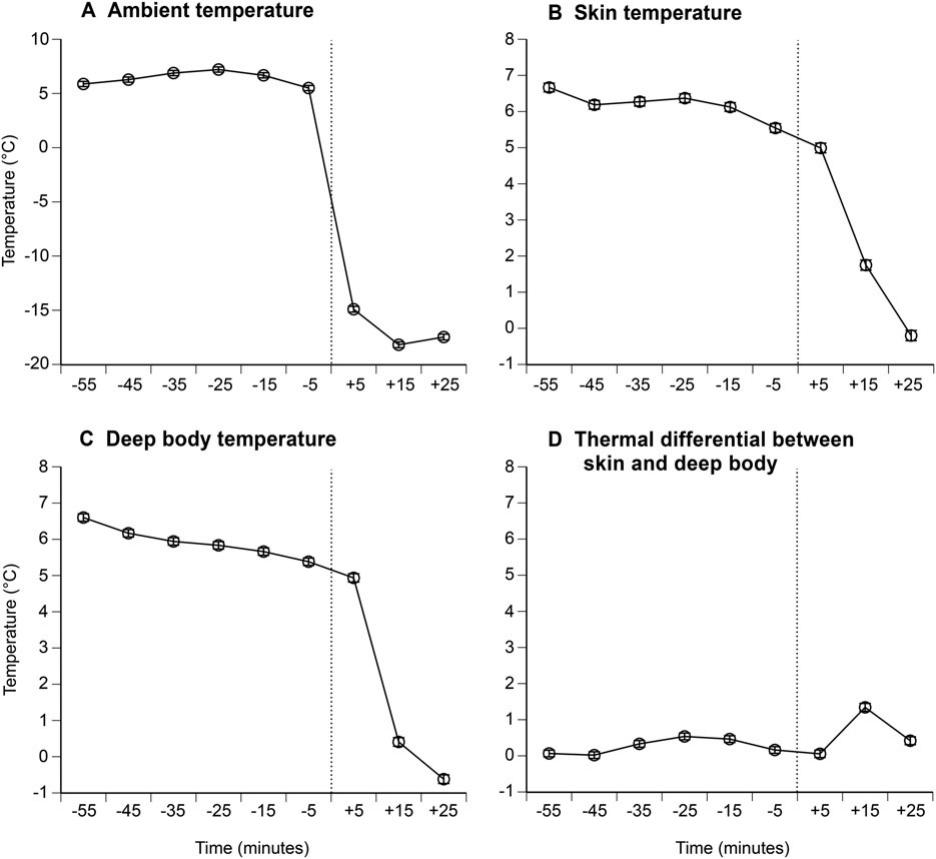
**Rates of cooling on the skin surface and inside the body for cane toads (*Rhinella marina*) transferred from a refrigerator to a freezer.** The panels show (A) ambient temperatures during the trials, (B) skin-surface temperatures, (C) deep-body temperatures, and (D) the thermal differential between the skin surface and the body core (means and standard errors). X-axis shows midpoint time for each 10-min period.

**Fig. 2.**
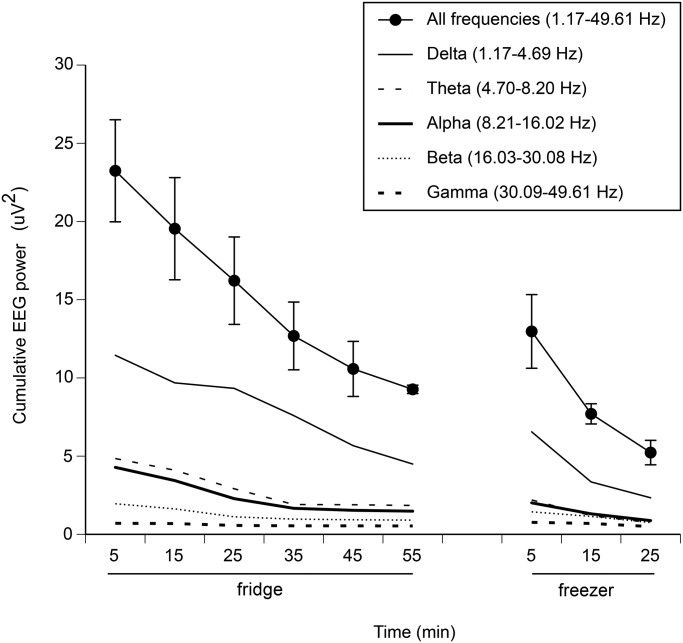
**Changes in brain activity measured as cumulative EEG power, and delta, theta, alpha, beta and gamma power, as a function of time spent in the new thermal regime.** Across all frequency bandwidths, brain activity shows a smooth decline across the first 60 min in the fridge and 30 min in the freezer (means and standard errors). X-axis shows midpoint time for each 10-min period.

A toad's limb and deep body temperature closely followed ambient temperature (Fig. 1A,B,C); differentials between the animal's skin and its core averaged <1°C (Fig. 1D). Accordingly, we recorded a continuous smooth decline in brain activity from fridge to freezer (Fig. 2). We saw no evidence of increased EEG activity, across any frequency bandwidths, as has been reported in animals exposed to painful stimuli (Lambooij et al., 2002; Zulkifli et al., 2014). Instead, the brain became electrically ‘quiet’ with decreasing temperature.

## DISCUSSION

Collectively, general features of the thermal dependency of nerve and brain function in ectotherms suggest that for cane toads (and potentially, for many species of amphibians and reptiles), cooling-then-freezing can offer a humane death. By the time that ice crystals form in peripheral tissues, the brain is almost as cold as those tissues; and hence, is unable to perceive or respond to nociceptive signals. Our experimental study on cane toads supported this more general result.

Clearly, there are caveats to this conclusion. First, it is important to pre-cool the animal before exposing it to freezing temperatures. Second, the potential for freezing to induce nociceptive signals will be greater for larger animals, which can maintain greater thermal differentials between the brain and peripheral tissues (Key, 2015), and have higher crystallisation temperatures (Lee and Costanzo, 1998). However, the toads that we used (>200 g) were far larger than adults of most amphibians and reptiles. Mean adult mass >100 g occurs in <5% of amphibian species and <10% of lizard species (Pough, 1980), so our results have broad applicability. Third, low temperatures may not suppress nerve impulses as effectively in cold-adapted species, or cold-acclimated individuals, as they do in tropical taxa (Key, 2015). Species-specific studies are essential before applying hypothermia to kill individuals of any amphibian or reptile taxa that are routinely active at low body temperatures.

Despite these caveats, our review of published literature and experiments on cane toads suggest that for many ectotherms (especially small-bodied, warm-climate taxa), cooling then freezing offers a humane form of euthanasia. Unfortunately, this simple, readily-accessible method is currently outlawed internationally by ethics committees: not because of contrary evidence, but because of speculation combined with a lack of critical analysis of available literature, and a dearth of empirical research. Similar problems extend to many other ethics issues (Langkilde and Shine, 2006). There is no point blaming the members of ethics committees; they are doing the best job they can, but cannot be expected to evaluate primary research literature or conduct their own experiments. We urgently need researchers to take up the challenge of clarifying which methods are humane, and which are not. Until scientists provide that evidence, animals will continue to suffer unnecessarily.

## MATERIALS AND METHODS

We implanted electroencephalogram (EEG) electrodes for recording brain activity in four wild-caught adult female cane toads (115–133 mm snout-urostyle length, 201–235 g). After the animals were anaesthetised with MS 222 (tricaine methanesulfonate; 3 g/l), we drilled four holes (0.5 mm diameter, two per cerebral hemisphere) through the exposed cranium to the level of the dura overlying the dorsal cortex. A fifth hole was drilled over the olfactory bulbs for the ground. All electrodes were gold-plated, round-tipped pins (0.5 mm diameter) glued in place using cyanoacrylic adhesive. Electrode wires (AS633, Cooner Wire, Chatsworth, California, USA) terminated at a connector fixed on the head with two stainless steel screws (25/095/0000, Hilco, London) and light-curing dental acrylic (Dentsply, Mt Waverley, Victoria). Electrode position was verified by dissection at the end of the study. Toads were then transferred to a damp cage, monitored until they regained normal motor function, and allowed a 10-day period of post-operative recovery at 30°C with food and water available *ad libitum*. EEG activity was recorded at 100 Hz using a head-mounted, miniature (25×25×9 mm) and lightweight (8 g, including battery) Neurologger 2A datalogger (Vyssotski et al., 2009; Lesku et al., 2012).

To record body temperatures during cooling and freezing, we inserted a thermocouple wire subdermally into each toad's left hind limb (to measure temperature immediately below the skin) and into the cloaca (to measure deep body temperature), respectively. These thermocouple leads, as well as one measuring ambient temperature, were connected to a TC-2000 thermocouple meter (Sable Systems, Las Vegas, NV USA) and logged each minute using ExpeData software via a UI2 analogue/digital converter temperature logger (Sable Systems, Las Vegas, NV USA). Prior to experiments, toads were transferred into a Faraday cage (22×17×12 cm) then placed into a standard household refrigerator (Kelvinator, Charlotte, NC USA). Once the toad's core reached fridge temperature (∼5°C), it was transferred to a household freezer (Fisher and Paykel, Auckland, New Zealand) for 30 min. Toads removed from the freezer after this time were dead (did not regain consciousness).

Fast Fourier Transforms were performed on 4-s, artefact-free epochs to calculate power in 0.39 Hz bins between 1.17 and 49.61 Hz using RemLogic 3.2 software (Embla Systems, Broomfield, CO USA). Cumulative EEG power was calculated as a measure of brain activity in 10-min bins, starting at the time placed in the new thermal regime. We also quantified power in the bandwidths typically used in analysis of the mammalian EEG (delta, theta, alpha, beta and gamma) and recently applied to amphibians (Fang et al., 2012).

All procedures were approved by the University of Wollongong Animal Ethics Committee (protocol no. AE10/05).
